# Expression of HMB45, MelanA and SOX10 is rare in non-small cell lung cancer

**DOI:** 10.1186/s13000-018-0751-7

**Published:** 2018-09-11

**Authors:** Mark Kriegsmann, Katharina Kriegsmann, Alexander Harms, Rémi Longuespée, Christiane Zgorzelski, Jonas Leichsenring, Thomas Muley, Hauke Winter, Daniel Kazdal, Benjamin Goeppert, Arne Warth

**Affiliations:** 1 Institute of Pathology, University Hospital Heidelberg, Im Neuenheimer Feld 224, Heidelberg, Germany; 2 Department of Internal Medicine V, Hematology, Oncology and Rheumatology, University Hospital Heidelberg, Heidelberg, Germany; 3 Translational Lung Research Centre Heidelberg, Member of the German Centre for Lung Research, Heidelberg, Germany; 4 Translational Research Unit, Thoraxklinik at Heidelberg University, Heidelberg, Germany; 5 Department of Thoracic Surgery, Thoraxklinik at Heidelberg University, Heidelberg, Germany; 6 Present address: Institute of Pathology, Cytopathology, and Molecular Pathology, UEGP, Gießen, Wetzlar, Limburg, Germany

**Keywords:** NSCLC, Lung cancer, SOX10, HMB45, MelanA, Immunohistochemistry

## Abstract

**Background:**

Non-small cell lung cancer (NSCLC) and melanoma are frequent entities in routine diagnostics. Whereas the differential diagnosis is usually straight forward based on histomorphology, it can be challenging in poorly differentiated tumors as melanoma may mimic various histological patterns. Distinction of the two entities is of outmost importance as both are treated differently. HMB45 and MelanA are recommended immunohistological markers for melanoma in this scenario. SOX10 has been described as an additional marker for melanoma. However, comprehensive large-scale data about the expression of melanoma markers in NSCLC tumor tissue specimen are lacking so far.

**Methods:**

Therefore, we analyzed the expression of these markers in 1085 NSCLC tumor tissue samples. Tissue microarrays of NSCLC cases were immunohistochemically stained for HMB45, MelanA, and SOX10. Positivity of a marker was defined as ≥1% positive tumor cells.

**Results:**

In 1027 NSCLC tumor tissue samples all melanoma as well as conventional immunohistochemical markers for NSCLC could be evaluated. HMB45, MelanA, and SOX10 were positive in 1 (< 1%), 0 (0%) and 5 (< 1%) cases. The HMB45 positive case showed co-expression of SOX10 and was classified as large cell carcinoma. Three out of five SOX10 positive cases were SqCC and one case was an adenosquamous carcinoma.

**Conclusions:**

Expression of HMB45, MelanA and SOX10 is evident but exceedingly rare in NSCLC cases. Together with conventional immunomarkers a respective marker panel allows a clear-cut differential diagnosis even in poorly differentiated tumors.

## Background

Non-small cell lung cancer (NSCLC) and melanoma are frequent entities in pathological routine diagnostics. Whereas the differential diagnosis is often feasible based on histomorphology alone, especially in resection specimens or when melanin pigment is evident, the distinction of both can be challenging in small biopsies or cytology preparations. This is especially true for cases with undifferentiated morphology as melanoma may mimic various histological patterns [1]. Although, a tumor situated in the lung is more likely to be lung cancer, the lung is a frequent site of metastatic spread especially in patients with NRAS-mutated melanoma [2, 3] and primary melanoma of the lung is recognized in the literature as well [4–6]. Distinction of the two entities is of outmost importance as both are treated differently [7, 8]. Of note, the common BRAF mutations in melanoma (usually V600E) [9] can be found in pulmonary adenocarcinomas (usually non-V600E) [9–11]. Commonly applied immunohistological markers in this scenario are S100, Human Melanoma Black (HMB45), melanoma antigen recognized by T cells 1 (MelanA), SRY-related HMG-box 10 (SOX10), cytokeratin 5/6 (CK5/6), NapsinA, p63 (p40) and thyroid transcription factor-1 (TTF-1) [12, 13]. While the expression of CK5/6, NapsinA, p63 and TTF-1 has been extensively studied in NSCLC [14, 15] and the immunoreactivity of S100, HMB45, MelanA and SOX10 is well described for melanoma [16], the comprehensive expression of melanoma markers in NSCLC has not been analyzed in a large NSCLC tumor tissue cohort to date, except for S100 [17]. This is surprising as especially SOX10 has prompted recent interest and has been reported in various other cancer entities. Therefore, we systematically analyzed the expression of HMB45, MelanA, SOX10, CK5/6, NapsinA, p63 and TTF-1 in 1027 NSCLC cases including 498 adenocarcinomas (ADC), 424 squamous cell carcinomas (SqCC), 44 adenosquamous carcinomas (ADSqCC), 51 large cell carcinomas (LC) and 10 pleomorphic carcinomas (PC).

## Methods

### Cohort characteristics

Formalin-fixed and paraffin embedded NSCLC tumor tissue specimens resected from 2004 to 2007 in the Thoracic Hospital Heidelberg were extracted from the archive of the Institute of Pathology, Heidelberg University, with the support of the tissue bank of the National Center for Tumor Diseases (#2508, ethics committee University of Heidelberg #S-205). Tissues were used in accordance with the ethical regulations of the NCT tissue bank defined by the local ethics committee and according to the Declaration of Helsinki. Diagnoses were made according to the recommendations of the World Health Organization classification for lung cancer 2015 [12]. A cohort of 1085 NSCLC cases was selected. Tissue microarray construction was done as described previously [15, 18, 19]. The results from the conventional NSCLC markers CK5/6, NapsinA, p63 and TTF-1 were stained and published previously [15]. A detailed description of the clinical characteristics of the NSCLC tumor tissue cohort is provided in Table 1.

**Table 1:** Clinical characteristics of the cohort

Patients, *n*	1027
Median age, years (range)	64 (30–85)
Patient gender	n (%)
Male	734 (71)
Female	293 (29)
Histology	
ADC	498 (48)
SqCC	424 (41)
ADSqCC	44 (4)
LC	51 (5)
PC	10 (1)
TNM-Classification	
pT1a	84 (8)
pT1b	114 (11)
pT2a	484 (47)
pT2b	170 (17)
pT3	155 (15)
pT4	20 (2)
pN0	503 (49)
pN1	244 (24)
pN2	249 (24)
pN3	6 (1)
pNX	25 (2)
pM1	25 (2)
pM0	1002 (98)
Clinical stage	
IA	133 (13)
IB	255 (25)
IIA	209 (20)
IIB	105 (10)
IIIA	286 (28)
IIIB	14 (1)
IV	25 (2)

*ADC*, adenocarcinoma, *ADSqCC*, adenosquamous carcinoma, *LC*, large cell carcinoma, n, number, *SqCC*, squamous cell carcinoma

### Immunohistochemistry

**Fig. 1:**
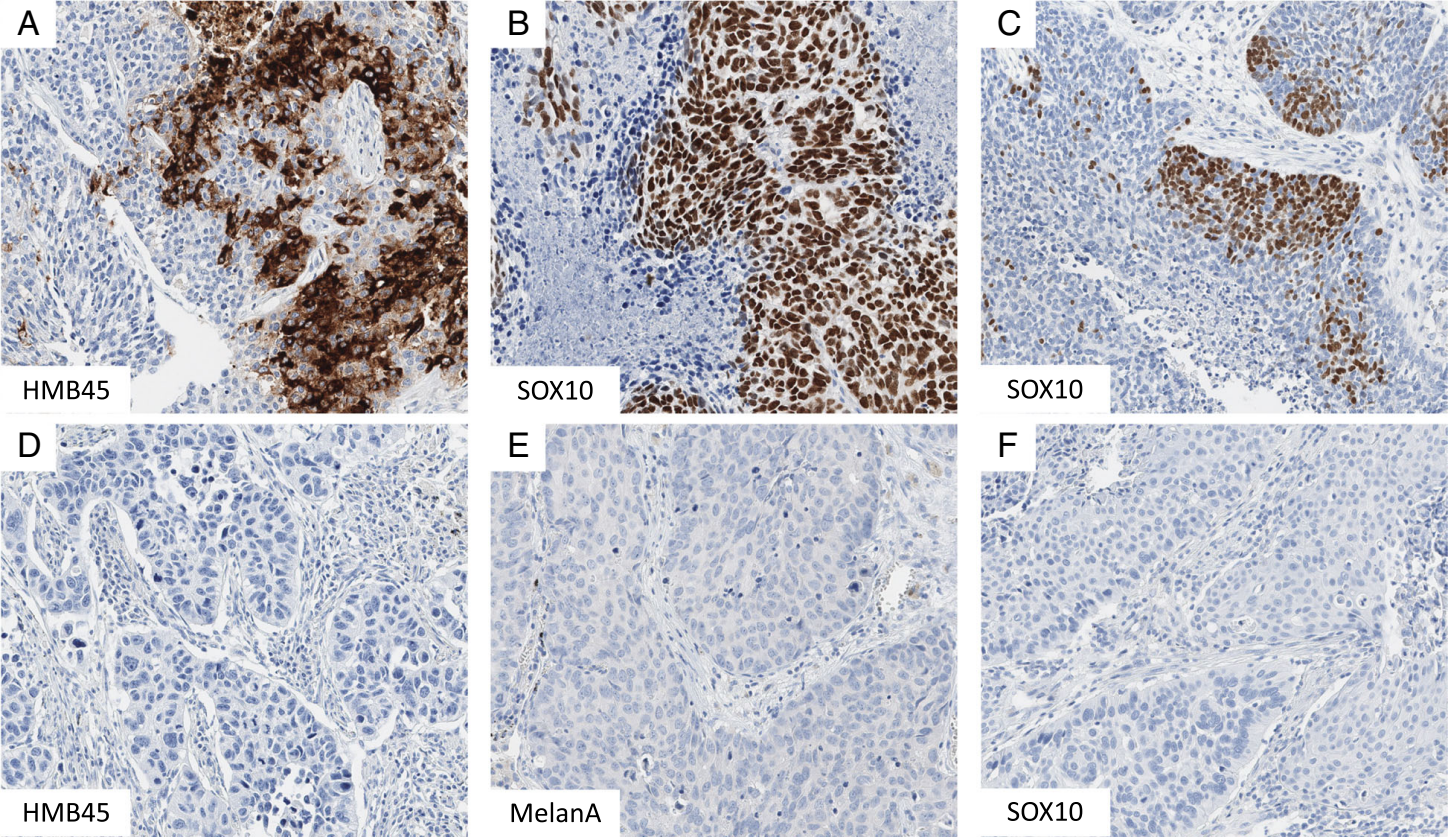
Examples of immunohistochemical stainings. Positive (**a**-**c**) and negative (**d**-**f**) examples of HMB45 (**a**, **d**) and SOX10 (**b**, **c**, **f**) are displayed. A representative image of a negative MelanA stain (**e**) in SqCC is also demonstrated. SOX10 expression reached from strong expression in all tumor cells (**b**) to focal positivity in only a subset of cells (**c**). Mag.: 200×. HMB45, Human Melanoma Black 45, SqCC, squamous cell carcinoma, SOX10, SRY-related HMG-box 10 protein

Immunohistochemical staining was performed as previously described [20]. In brief, slides were deparaffinized, pre-treated with an antigen retrieval buffer and stained using an automated device. Conventional NSCLC markers were stained on a Techmate 500plus (Dako, Hamburg, Germany) and melanoma-markers on a Ventana Benchmark Ultra (Roche, Rotkreuz, Switzerland). The antibody and staining characteristics are shown in Table 2. Staining was evaluated based on positivity of > 1% positive tumor cells by an experienced pathologist (M.K.). The cut-off of 1% positive tumor cells was chosen to avoid classification of minimal staining (which would likely be background staining) as positive. Typical examples of positive and negative samples of the respective antibodies are shown in Fig. 1.

**Table 2:** Antibodies used in this study and staining conditions

Antibody	Company	Clone	Pretreatment	Buffer incubation time (min)	Antibody incubation time (min)	Dilution
CK5/6	Dako	D5/16 B4	Tris/Borat/EDTA, pH 9	56	24	1:50
HMB45	Dako	HMB45	Tris/Borat/EDTA, pH 8.4	64	24	1:75
MelanA	Ventana	A103	Tris/Borat/EDTA, pH 8.4	64	24	RTU
SOX10	Cell Marque	EP268	Tris/Borat/EDTA, pH 8.4	48	24	1:100
Napsin A	Novocastra	IP64	Tris/Borat/EDTA, pH 6	32	24	1:400
p63	DCS Immunoline	4A4	Tris/Borat/EDTA, pH 6	40	24	1:25
TTF-1	Novocastra	SPT24	Tris/Borat/EDTA, pH 9	56	24	1:200

*CK*, cytokeratin, *HMB45*, Human Melanoma Black 45, *SOX10*, SRY-related HMG-box 10 protein, *TTF-1*, thyroid transcription factor-1

### Data analysis and software

All statistical analyses were done in R-Statistical Software (www.r-project.org, v.3.4.2, Free Software Foundation) and R-Studio (v.1.1.383, Affero General Public License, Boston, USA). The Figure was created in Inkscape (v. 0.92) and Powerpoint (Microsoft, Redmond, USA).

## Results

### Evaluation of tissue microarrays

In a total of 1027 NSCLC tumor tissue specimen HMB45, MelanA, SOX10, CK5/6, NapsinA, p63, and TTF-1 could be evaluated. In the other cases one or more of the conventional stainings (CK5/6, NapsinA, p63 and TTF-1) could not be analyzed due to floating or rolling of tissue cores. The drop-out rate was 5%. All neglected cases were negative for HMB45, MelanA and SOX10.

### Expression of conventional NSCLC markers CK5/6, NapsinA, p63, and TTF-1

CK5/6, NapsinA, p63 and TTF-1 were positive in 457 (44%), 409 (40%), 510 (50%) and 502 (49%) cases, respectively. While CK5/6 and p63 were most commonly expressed in SqCC (403 (95%) and 405 (95%)), NapsinA and TTF-1 were mainly positive in ADC (371 (74%) and 436 (88%)).

### Expression of melanoma markers HMB45, MelanA, and SOX10 and interplay with conventional NSCLC markers

HMB45, MelanA, and SOX10 were positive in 1 (< 1%), 0 (0%) and 5 (< 1%) of cases respectively. The HMB45 positive case showed co-expression of SOX10. This particular case was also positive for AE1/3, CK7, CK5/6 (focal). NapsinA, TTF-1, p40, p63, CD56, synaptophysin, S100 as well as MelanA were negative. Based on morphology and immunohistochemistry this sample was classified as LC. All of the five SOX10 positive cases were also positive for CK5/6, three were positive for p63 and all were negative for NapsinA and TTF-1. Three out of five SOX10 positive cases were classified as SqCC, one was classified as ADSqCC based on the resection specimen. The other histological subtypes (ADC or PC) showed no expression of melanoma markers.

## Discussion

As novel targeted therapies for melanoma and NSCLC are available which show impressive effects, the differentiation of both entities is essential. However, little is known about the expression of melanoma markers in NSCLC tissue specimens, except for S100 [17]. In the present study we analyzed HMB45, MelanA, SOX10, CK5/6, NapsinA, p63, and TTF-1 in a large cohort of NSCLC cases and demonstrate that expression of melanoma markers is rare. A small subset of SqCC, LC and ADSqCC may be positive for SOX10.

HMB45 is a monoclonal antibody that has been first described in 1986 [21] and recognizes melanosomal glycoprotein gp100 (Pmel17). The anti-MelanA murine monoclonal antibody A103 [22] has been described in the late 1990s. Both are commonly applied in thoracic pathology for the detection of melanocytic tumors [2, 13], and to exclude rare thoracic tumors such as perivascular epitheloid cell tumors (PEComa) including lymphangioleiomyomatosis [23] or clear cell sugar tumor [24] as well as for the diagnosis of clear cell sarcoma of soft parts [25]. Besides its role in the differential diagnosis of thoracic tumors, HMB45 and MelanA expression has been demonstrated in sex-chord stromal tumors [26], t(6;11)(p21;q12)-translocation associated renal cell neoplasms [27], endometrial stromal sarcomas [28] and nerve sheet tumors [29].

A more recently described melanocytic immunomarker is SOX10 which regulates Wnt/β-catenin signaling, contributes to stem/progenitor activity and induces a mesenchymal transition expression [30, 31]. It has been suggested to be a useful marker for the differential diagnosis of melanocytic lesions [32, 33]. However, expression of SOX10 has also been recently described in benign adnexal skin tumors such as cylindroma and spiradenoma (uniformly positive) [34], schwannoma [33], in tumors of myoepithelial origin [33] and in a subset of malignant neoplasms for instance bladder cancer [35], breast cancer [36], ependymoma [37], gastric adenocarcinoma [38], hepatocellular carcinoma [39], nasopharyngeal carcinoma [40], ovarian tumors [41], prostate cancer [42], salivary gland tumors [43, 44], and squamous cell carcinoma of head and neck [33]. SOX10 positivity is usually restricted to a small subset of carcinomas (< 10%) except for triple-negative breast cancer that may more frequently express SOX10 [33].

To the best of our knowledge, HMB45, MelanA and SOX10 expression has not been evaluated in a large NSCLC cohort > 1000 samples so far and has been investigated only in a limited number of patients [45]. The later study included five normal lung samples and 25 neoplastic lung cancer samples, all of which were negative for SOX10. Miettinen et al. reported occasional SOX10 expression in SqCC of lungs and in one well-differentiated fetal pulmonary ADC, but they did not specify the number of positive SqCC cases, nor the overall number of lung cancer samples analyzed. In line with the expression of SOX10 in myoepithelial and basal cells, a subset of pulmonary SqCC have been described to be positive for the basal type cytokeratin CK15 [46]. However, the biological role of the expression of basal cell markers in NSCLC tissue specimen remains to be investigated. In our study we found only one HMB45 positive case classified as LC, which had co-expression of SOX10. SOX10 positivity was observed in five out of 1085 cases. Three of them were SqCC and one case was ADSqCC. None of the ADC or PC showed expression of melanocytic markers.

## Conclusions

In summary, we demonstrate in a large NSCLC tumor tissue cohort that expression of HMB45, MelanA, and SOX10 is exceedingly rare in NSCLC cases and almost restricted to tumors with squamous differentiation. Thus, together with the conventional immunomarkers for NSCLC, a marker panel including these stainings  is valuable in the differential diagnosis of thoracic neoplasms and melanoma and will lead to a definite diagnosis even in poorly differentiated tumors.

## Abbreviations

ADC: adenocarcinoma
ADSqCC: adenosquamous carcinoma
BRAF: v-Raf murine sarcoma viral oncogene homolog B
CK: cytokeratin
HMB45: Human Melanoma Black 45
LC: large cell carcinoma
MelanA: melanoma antigen recognized by T cells 1
NRAS: Neuroblastoma RAS viral oncogene homolog
NSCLC: non-small cell lung cancer
PC: pleomorphic carcinoma
PEComa: perivascular epitheloid cell tumor
SOX10: SRY-related HMG-box 10
SqCC: squamous cell carcinoma
TTF1: thyroid transcription factor-1
